# Clinical Lecture—Asthmatic Bronchitis—Follicular Pharyngitis

**Published:** 1888-12

**Authors:** Homer M. Thomas

**Affiliations:** Clinical Professor of Diseases of the Chest and Throat in the Woman’s Medical College, Chicago, Ill.; Physician and Surgeon for Diseases of the Throat and Chest, Central Free Dispensary, Chicago


### A CLINICAL LECTURE.

BY HOMER M. THOMAS, A. M., M. D.,

Clinical Professor of Diseases of the Chest and Throat in the Woman's Medical College, Chicago, Ill.; Physician and Surgeon for Diseases of the Throat and Chest, Central Free Dispensary, Chicago.

### ASTHMATIC BRONCHITIS.

Mrs. K. L., aged 38 and married, has had an affection of the chest for the last five years. Previous to that time her general health was good, her nutrition excellent, her chest free from any symptoms of disease. Coincident with the time that the present chest condition was discovered, she lived where she was subjected to the inhalation of a large amount of dust. The dust was that which comes from lime, the peculiar quality of lime which is used in cementing sewer joints. This lime was in the neighborhood where she worked, and she breathed it, from the fact that there were being destroyed barrels which had formerly contained the lime, and in chopping them up the dust from the lime barrels was carried into the air, and thence was respired by her. She noticed, at this time, first, the symptoms of severe cough, the cough lasting for only a short time and then ceasing. It became renewed whenever she was exposed to the inhalation of dust. She lived under these conditions for five months.

We have, then, as an exciting cause of this trouble, the direct infective irritation which resulted from the inhalation of lime dust. Now as to the process by which this lime dust can produce so much irritation. By the normal processes of respiration, the patient first respires the dust into the nasal passages, reaching the Schneiderian membrane which covers these passages, and excited irritation. This irritation gave rise to congestion; following the congestion there resulted hyperæmia; succeeding the hyperæmia there followed dryness, and from the stage of dryness there succeeded one of a marked serous exudate, and as a resultant cause from this stage of excessive secretion, there was a gradually diminishing secretion and then a return to the normal condition again. This is the pathology of the irritation, such as this patient suffered from, in its effect upon the Schneiderian membrane. But continuous with this membrane from the nose, there is a membrane which covers the structures known as the pharynx, larynx, trachea, and bronchial tubes. The primal irritation, as I have said, was first in the nose, then as an effect of this cause there resulted irritation, which affected the pharynx. This condition we would technically call a "follicular pharyngitis." Along with the follicular pharyngitis there was that of subacute laryngitis; succeeding that there was some local inflammatory action within the trachea, and after that there resulted a condition of acute bronchitis.

Now these successive processes of the disease were first acute in character, such as we all generally suffer from, and which we term a "common cold"—a cold in the head, throat or chest. But with the exciting cause persistent, as in this case, the patient was not able to eliminate the inflammatory state which existed in the head, throat and chest, hence the condition from repeated renewals of the exciting cause became chronic. The chronic condition has resulted in a typical illustration of the disease known as *asthmatic bronchitis.*

Now what do we understand by the term asthmatic bronchitis? It conveys to us a double meaning—that of a condition of bronchitis and asthma, hence the trouble is dual in its nature; the irritated condition of the bronchial mucous membrane gave rise to an inflammatory state of this membrane. That in turn gave rise to structural changes, which excited certain pathological states within the membrane; the bronchial mucous membrane, instead of having that white, glistening, smooth surface, characteristic of the healthy state, became engorged with blood, the engorgement led to a condition of hyperæmia, the hyperæmia in turn gave rise to excessive secretion, and the excessive secretion was succeeded by a condition of relaxation—by a softened state, as it were, of this membrane. This was followed by repeated inflammatory states, and that gave rise to a hypertrophy or thickened state of the membrane. With that condition present, there was profuse expectoration of a mucoid character, which was thrown off with difficulty. The expectoration was also slightly tinged with blood; but with the severe bronchitis present, the patient suffered from a complication resulting from the bronchitis, namely, a hypertrophy of the annular fibres of the bronchial tubes. They became tense and enlarged in their structure, and hence diminish the calibre of the right and left bronchial tubes. This diminution of the calibre of the tubes gave rise to impeded respiration, and the impeded respiration was succeeded by certain characteristic sounds which are diagnostic of this condition. Given a bronchial tube normal in size, free from any inflammatory exudate upon its surface, the passage of air in respiration is unattended with any difficulty. The normal sounds are free, clear, and are not even unmusical in their tone. However, with the condition of the hypertrophy of the annular fibres of the tubes, you can see that the passage of air would be arrested or impeded at points, because the enlargement of the tubes is not uniform; they may be constricted at one point and diminished at another.

Now the current of air in respiration, passing through these tubes, which are hypertrophied, gives rise to undue pressure upon them. This undue pressure of the current of air, in turn gives rise to certain whistling sounds, which are denominated râles, and the râles are of two types, the *sibilant* and *sonorous*.

Auscultation of this patient's chest reveals the sibilant and sonorous râles. Aside from the effects of this condition upon the bronchial tubes, we meet with another condition which is found in the partial obliteration of the capillary circulation of blood in the lungs. This capillary absence or impaired circulation is due to the very difficult respiratory efforts which are found necessary by patients subjected to asthmatic bronchitis. Their impaired action is occasioned by the extremely congested condition which the lungs suffer from during the paroxysmal periods of these attacks. With the impaired action in the chest, we have a hypertrophic condition of the heart. The hypertrophy of the heart in such cases is compensatory; it is but the effect of a cause, and hence is valuable and necessary. It is found in these cases mainly upon the right side of the heart, the right ventricle. This is so because of the difficulty of getting the circulatory current through the lungs. Were the condition arterial, the hypertrophy would be on the left side of the heart, but being mainly venous in its character, it is upon the right side to facilitate the circulation of blood through the pulmonary veins. The resultant hypertrophy necessarily gives rise to an accentuation of the cardiac sounds. The first sound of the heart is caused by the closure of the mitral and tricuspid valves. The hypertrophy occasions an accentuation of these sounds because of the difficulty of circulation of blood through the capillaries. The attendant cardiac hypertrophy can be demonstrated by percussion of the heart. There is also cardialgia present. These pains are more the result of an increased impact of the heart, or rather of an increased strength of the heart, its action upon the intercostal nerves giving rise to irritation from this increased action or unduly strengthened state. With this condition of hypertrophy there are certain structural changes which result within the ventricle of the heart. The connective tissue, which is a part of the muscular fibre within the heart, is increased in size through the process of hyper-nutrition, this hyper-nutrition resulting in the condition which gives rise to the typical hypertrophy that exists in this case.

After considering in this way the active or exciting cause of this condition, we are at a loss to explain why, since the removal of the patient from the exciting cause, the condition has still existed. I think one explanation is found in the increase of weight the patient has suffered from. She is getting heavier and stouter as her age increases, and with that condition there is necessarily a strained action upon the chest, both upon the lungs and upon the heart, for with this increased weight there is difficulty of locomotion; there is attendant shortness of breath, and there are many subsequent symptoms that follow increased weight without compensating strength. The tendency of asthmatic bronchitis is to continue itself when once established, even if the exciting cause is but slight. This case is typical in not having the trouble during the summer. Climatic causes are the explanation of this. With the advent of the fall and winter months, and their consequent severe changes of temperature, the patient suffers greatly because of one natural fact in philosophy, namely, that the tendency of cold is to contract; the reverse, that of heat, being to expand, would explain why in summer she is free from this trouble more so than in winter. During winter the excessive cold, the dampness, the sudden changes in the atmosphere, all lead to a determination of blood to these parts; this in turn leads to a congestive state of the membrane, and following this there is a constricted condition of the chest, the effect of atmospheric changes upon the bronchial mucous membrane.

In the line of remedial agents for the relief of this trouble, what can we suggest? First, there is the immediate dyspnœa to be relieved, and it can be accomplished in several directions. In some conditions like this, the causative factor is found within the congested state of the Schneiderian membrane in which it approximates itself upon each side and in which the edges are contiguous. The effect in the chest, then, is but a result of the nasal condition, and such troubles are relieved marvelously by the hydrochlorate of cocaine. A combination containing 1 gr. bi-carbonate of soda, 1 gr. biborate soda, 3 grs. sulphate magnesia, 4 grs. cocaine and enough sugar of milk to make 100 grains is a very pleasant and useful way in which to administer this medicine. It is administered to the membrane by insufflation; the quantity used at a single time should not exceed that represented by the quarter of an inch of the powder within an ordinary size glass tube used for insufflation. This, when blown into the membrane, relaxes the spasm of the parts, is astringent in its effects, anodyne as well, and frees the nasal tract at once, and hence relieves the chest where the trouble is central within the nasal passages. But such is not the case in the patient before you. Here the condition is one more central in the chest. During the immediate paroxysm of dyspnœa that results during her attacks, the most useful combination I have tried is that which consists of one-eighth of a grain of sulphate of morphia, 7 ½ grains of the hydrate of chloral, given at one dose, to be repeated every half hour or an hour as needed to relieve the dyspnœa. Then, in order to free the bronchial mucous membrane from secretion, you want to use remedies that have a sedative action upon this membrane and are expectorant in their type. A useful combination might consist of syrup of Squill's, the carbonate of ammonium, glycerine, camphorated tincture of opium in syrup of liquorice.

Now is it necessary to do anything for the cardiac condition, which is the sequela of the cardiac state ? No. Warm clothing is something that is very often neglected in such patients. The chest should be very warmly covered; there should be no possibility of cold striking the sensitive bronchial mucous membrane. The feet should be kept dry, the bowels open, and the processes of nutrition looked after, so that the strength of the patient may be conserved and assisted.

### FOLLICULAR PHARYNGITIS.

The next case which I present to you is Mrs. H., age 34 years, American. Her trouble is known as *follicular pharyngitis*, complicated by a scrofulous diathesis. At the age of 10 years she suffered from whooping cough, and is inclined to think that her throat has been affected ever since. Her trouble is aggravated by residence in Chicago, where she has lived for the last eight years. Her appetite is variable; she seldom has coughs; she suffers from a severe catarrhal discharge from the throat on exposure to dampness and cold.

Now, the relation that the scrofulous condition may have to that of the evident *follicular pharyngitis* is of interest. To what extent may the condition of scrofula be an exciting cause to the *follicular pharyngitis?* We can only, in the most general way, say that they are related. We have nothing in the pathology of scrofula or in the pathological state of *follicular pharyngitis* which directly relates to the one as causative of the other.

We can only reason by analogy that any condition like scrofula, in which depraved nutrition, weakened vitality, an innervated state of the nervous system in which the blood is not sufficiently nutritive, may give rise to many conditions which would be the outgrowth of a depraved or vitiated constitutional state, and hence the relation that may exist between these two troubles is more a matter of inference than of a direct pathological sequence. However, it is likely that the severity of the *follicular pharyngitis* is markedly increased by the scrofulous diathesis, which is present. It is extremely probable that the one intensifies the other.

Now, as to the *follicular pharyngitis* which exists in this case. Its antecedent cause was a common cold which came about through exposure, and which gave rise to much the same set of symptoms as noted in the previous case. Following the simple cold in the head, as we would commonly designate it, there resulted hyperæmia of the pharyngeal membrane. This hyperæmia or blood engorgement gave rise to dryness, the dry state being followed by one of excessive secretion or inflammatory exudate. The exudate gave rise to certain mucous patches in the pharyngeal membrane. This appearance is designated as *follicular* from the *follicular*-like type which is shown upon examination. These follicles are filled with mucus, which become dry, hardened and scattered over the pharyngeal membrane. They exist in patches and are found upon the posterior surface of the pharynx. The mucus is hard, dry and viscid in quality, and gives rise upon the wall of the pharynx to certain punctated or pin-pointed projections. The tendency of this state is to produce cough, and the cough dislodges the secretion which is accumulated, only to be followed again by more of the same type. This renewed type of secretion gives rise to a continued irritation within the wall of the membrane, and the irritable state in turn from an acute type succeeds to a chronic one. The chronic condition gives rise in time to a relaxed state of this membrane, a catarrhal pharyngitis, and it is essentially the condition which is known as clergyman's sore throat—a trouble which speakers, singers, and those using the voice much in speaking, suffer from.

This follicular state of the pharynx may be aggravated by gastric disturbances of the stomach. The causative relation existing between a chronic dyspepsia and the *follicular pharyngitis* is such as would relate the pharyngeal membrane to irritant gases which would be belched up by a patient suffering from an irritative form of dyspepsia. The process of irritation that would follow such a state of the stomach would reflexly affect the throat, and this gaseous action upon the throat from the fermentation of particles of food would give rise to acute pharyngitis, and that in turn might become a follicular or chronic pharyngitis. This case in point is dual in its causative relation then. There exists an atonic dyspepsia and post-nasal catarrh. The atonic dyspepsia irritates the membrane probably 50 per cent. and the post-nasal catarrh is responsible for the remaining 50 per cent.

What are you going to do to relieve such a patient? First, you want to look to the gastric state; that is, primarily, where the condition of *follicular pharyngitis* is dual, for with the atonic dyspepsia you have a depraved nutrition, and with a defective, insufficient nutrition you must have a lack of vitality or reconstructive energy in the system. This would make possible repeated colds, or likely the existence of pharyngitis from a gastric disorder; hence the tone of the stomach, the vitality and integrity of its action should be conserved, and the processes of nutrition made so perfect that this cause of the trouble would be removed.

As to the local treatment of this condition. It should look to the removal of the catarrhal state in the naso-pharynx. That is best assisted by the use of agents which tend to stimulate this passage and relieve it of its abnormal secretions. The local use of douches, which contain bicarbonate of soda, benzoate of soda, eucalyptol, thymol, glycerine and alcohol, in combination are very useful. They free the membrane of its excessive secretion, diminish the hyperæmia of the parts and make the local application of medicines more effective. Having secured a clean state of the membrane lining the naso-pharynx through the use of these douches, the selection of some powder for local application is necessary. The indications to be met by the use of the powder in insufflation are those which will both stimulate and antisepticize the passage, and a powder consisting of equal parts of iodol and pulverized boracic acid is very useful to meet these indications. Then, after having secured freedom from irritant masses above the naso-pharyngeal trouble, the local treatment of the pharynx itself is indicated. One of the most efficient stimulating applications that can be used is that of a 10 per centum solution of the nitrate of silver. That tends to free the pharynx of its diseased secretions and also has a markedly stimulating effect upon the membrane itself.

In the way of preventive measures for this condition, whatever contributes to the general health of the patient and eliminates possible causes of colds are indicated and advised. The better the constitutional state the more efficient the resisting powers of the patient, so that the effective relief of this condition is that which is found in both constitutional and local medication.

---

DIPHTHERIA CARRIED BY A TURKEY.—A fowl with diphtheria was brought to the house of a veterinary surgeon on April 24 and died on the 29th. The feeding and nursing of the bird devolved on a lad, aged 14, who was assisted by his brother, aged 5. On the evening of May 11, the writer was called to see the little boy of 5, who had been poorly for a day or two. He had enlarged cervical glands on the left side, which had come on rapidly. He was a delicate little fellow, with fair hair and anæmic aspcct. The fauces were more or less covered with diphtheritic membrane, the left tonsil more especially. Under the administration of biniodide of mercury and iron, the throat symptoms cleared up and the child made a good recovery. On the day after this case was first seen, the boy who fed the fowl was very feverish and had similar patches over his fauces, but not to the same extent as his brother. A sister, aged 9, had also a similar explosion on the fauces. On the 18th, the mother, who nursed them, was attacked and was similarly treated. They were all kept well up with beef-tea and stimulants.—*British Medical Journal.*

